# Raynaud's Disease

**Published:** 1912-09

**Authors:** David A. Alexander


### RAYNAUD'S DISEASE.

BY

DAVID A. ALEXANDER, M.B., Ch.B.Vict.

WE went in to luncheon. Something was said of our friend —— and the new-found malady, Raynaud's disease.

*J. M.*—Joseph de Maistre says that in the innocent primitive ages men died of diseases without names.

*Mr. G.*—Homer never mentions diseases at all.

*J. M.*—Not many of them die a natural death in Homer.

*Mr. G.*—Do you not recollect where Odysseus meets his mother among the shades, and she says :—

> Οὔτε τις οὖν μοι νοῦσος ἐπήλυθεν . . .
> ἀλλά με σός τε πόθος σά τε μήδεα, φαίδιμ' 'Οδυσσεῦ,
> σή τ' ἀγανοφροσύνη μελιηδέα θυμὸν ἀπηύρα.

> It was not sickness that came upon me ; it was wearying for thee
> And thy lost counsels, glorious Odysseus, and for all thy
> Gentle kindness, this it was that broke the heart within me.

*J. M.*—Beautiful lines. Πόθος such a tender word, and it is untranslatable.

*Mr. G.*—Oh, *desiderium.*

> Quis desiderio sit pudor aut modus
> Tam cari capitis.

> What shame or limit should there be
> In longing for so loved a friend.

*J. M.*—The Scotch word "*wearying*" for somebody. And *Sehnsucht.*

Morley, *Life of Gladstone,* vol. iii., date Dec. 17th, 1890.

A. F. is a tailoress of 38. In February, 1911, she complained of an intractable ulcer on the extremity of a toe, which had been regarded as a broken chilblain. After treating it for two or three weeks with no success, I chanced one day to observe her fingers blanch, and suspected that she was a sufferer from Raynaud's disease, and that the key to her condition was the state of the vaso-motor system.

She is of a neuropathic stock. An uncle had attacks of insanity from an emotional cause—grief at the sudden death of a son. The members of her own family show for each other an anxiety and solicitude which is almost morbid. At the age of twenty—seventeen years past—she sustained a shock in the death of her father. To the fact that "she nor spake nor uttered cry" do her friends attribute the development directly afterwards of epilepsy.

She is of fully middle height, big boned, thin, with a sunken aspect, a pensive and rather hungry look. The complexion is usually red and dusky ; the skin moist and warm when not under the influence of her affection, resembling that of other members of the family ; the pulse is regular and soft and of low tension ; the arteries unthickened and resilient ; the eyes are prominent ; the thyroid unenlarged ; the digital joints unthickened ; the fingers attenuated, and the skin over them thin and papery. The features of Raynaud's category have shown themselves at different times.

Local syncope.—In genial weather and when the complaint remits little is to be remarked. Often, however, there is a marbling or mottling of the hands, noticeable in itself, and not to be overlooked when the intenser vascular derangements have been once seen. It is the fingers and toes which local syncope affects. The onset is without pain ; the attacks are succeeded by numbness and tingling. They are inconstant and fleeting ; their duration seldom an hour, usually to be told in minutes. I had often seen the patient before observing the first, and she might readily have continued to exhibit one complaint or another without the peculiarity revealing itself as the nexus and explanation of them all. In his work on the subject Monro descants upon the fugacious character of the attacks, and also the permanent effects of the disease comprised in a single occurrence of spasm.

Local asphyxia.—This accompanies or follows the preceding, and the contrast between purple and white is then conspicuous. On a January evening, when the patient was introduced to a clinical meeting, the coldness of the night brought out these features, for she had come unprovided with the mittens or goloshes, or that extra clothing such sufferers are advised to wear. During adolescence she had indeed been liable to cold feet and to chilblains, but not as she is now to "blueness" and deadness of the fingers.

Local gangrene.—It was the third left toe where this appeared. The extremity was affected, not the dorsum as is usual in chilblain. The end of the toe looked as if it had been bruised, and eventually presented a gangrenous ulcer ; pain was great, and from the toe radiated up the calf, but there were no symptoms of neuritis. As the patient's condition improved under rest and care and opium, the nature of the lesion became clearer ; a fragment of skin sloughed off, owing to occlusion of its vessels, and a cicatrix formed, which still marks the area. It is true that in this case there was no corresponding affection on the right side to illustrate the symmetry of this disease. Perhaps it is also significant that at this time on the left calf was a recrudescence of eczema, which had broken out seven years before. There was none of the cuticular shredding which characterises eczema rubrum, and after recovery the skin was seen to be thinned and atrophic, and with here and there a small underlying nodosity.

Skin affections.—Of erythema nodosum (says Malcolm Morris) the pathology is that of hyperæmic erythema. Local vaso-motor disturbance is followed by inflammatory effusion of fluid and escape of white blood corpuscles. Seven years ago the patient had a severe attack of "rheumatic lumps," and I

take the small nodosities under the skin to be their vestiges. She has recently undergone another, at the age of nearly forty, when such outbreaks are uncommon. The nodes appear on shins and knees, a few on the arms; they are larger than is usual; are tender and very painful; they leave as they pass some purpuric discolouration. When the patient stands the legs became œdematous and cyanotic. There is no joint effusion nor cardiac affection. The first week was pyrexial.

It will be recalled that French and German writers recognise two affections as peculiar to Raynaud's disease. *Regional ruber*, in which isolated spots appear, red, like fuchsin, resembling a raspberry, with vessels injected, with elevation of temperature (Weiss). *Tachetes*—reddish-blue or purple patches, not removable by pressure, sometimes very persistent, due to extravasation of blood in the deeper folds of the skin.

Paresis.—One transient attack illustrated in this case the liability in Raynaud's disease to functional paralyses. It fell during the incidence of the erythema, while the patient was keeping her bed. The right arm became from the shoulder downwards helpless, numb and blue. Such was the account given by the patient and her friends. They could state no exciting cause. It neither preceded nor followed one of her fits, nor had it any of the stigmata of *petit mal*. When she was seen there remained only a relative feebleness in the grasp of the right hand.

With this seizure may be mentioned ocular signs. Certain branches of the retinal arteries are narrow. Students of epilepsy have failed to establish a constant relation between the calibre of the optic vessels, the state of the pupils and the epileptic outbreaks. In Raynaud's disease, on the other hand, a spasm of the arteries has been demonstrated during the prevalence of partial amaurosis. The claudication to be detected here, however, has never given rise to any symptoms.

Epilepsy.—Sir William Gowers asserts that if we are to attribute epilepsy to a shock it should occur before the lapse of a week. I cannot find in the case of this patient how many days passed between her father's death and her first seizure. In the minds of her friends the events are bound together, and with them all her subsequent ill-health also. The attacks have no settled periodicity; most are nocturnal; three weeks rarely pass without a recurrence. As described, they conform to true epilepsy. There is no aura; the somnolence and peevishness which follow are characteristic.

It might be suspected that the bromide the patient takes retards her general betterment. Massive doses never have been given, and strontium sometimes instead of potassium. The cutaneous affections were not complicated by a bromide rash. On the other hand, one admits a diffidence with the drug in a case where general nutrition is so much called for, and by which alone it might be hoped the attacks would be cured. Epileptiform seizures happen so often with a Raynaud's phenomenon that a common cause must be allowed to them, although we are now forbidden, with Kussmaul and Tenner, to derive epilepsy from spasm of cerebral vessels, and taught by Hughlings Jackson to assign it directly to the cells of the cortex.

Hemorrhage.—On two occasions œdema of the lungs has followed a convulsion, respiration being rapid, breathing harsh, rales everywhere audible, expectoration copious and watery. But the latter was also stained with blood, and not from any such local injury as a bleeding tongue, for it uniformly tinged the frothy sputum. To the friends who knew the significance of hæmoptysis this was a positive proof of a decline. On neither occasion, however, could T.B. be discovered. Expectoration ceased in a day, leaving no local sign of any pulmonary lesion. And should tuberculosis supervene, as it may, I should still regard this feature as a pneumostaxis, and comparable to paroxysmal hæmoglobinuria, although I did not determine whether in the sputum the red cells were intact or not.

Of other symptoms little is to be remarked. There is no osseous atrophy nor digital shortening, albumen is never found in the urine, menstruation is irregular. Digestion is most feeble; for milk there is a standing aversion, and this is very unfortunate when nutrition is essential and calcium might prove specific. Calcium chloride as a drug was without effect. Opium has served well in time of pain, and not vaso-dilators, but adrenalin seemed to coincide with a period of betterment.

The prognosis in Raynaud's disease is not invariably bad, and the patient's present debility ought not to deny her the rallying influence of hope. Time may weaken the fretting of the emotions and the perturbations of the vaso-motor system may then cease, a stable physiological state be regained, diet resumed, and the malady and its name be forgotten.

